# Impacts of Perinatal Dioxin Exposure on Motor Coordination and Higher Cognitive Development in Vietnamese Preschool Children: A Five-Year Follow-Up

**DOI:** 10.1371/journal.pone.0147655

**Published:** 2016-01-29

**Authors:** Nghi Ngoc Tran, Tai The Pham, Kyoko Ozawa, Muneko Nishijo, Anh Thi Nguyet Nguyen, Tuong Quy Tran, Luong Van Hoang, Anh Hai Tran, Vu Huy Anh Phan, Akio Nakai, Yoshikazu Nishino, Hisao Nishijo

**Affiliations:** 1 Department of Epidemiology and Public Health, Kanazawa Medical University, 1–1 Daigaku, Uchinada, Ishikawa, 920–0293, Japan; 2 Ministry of Health, Vietnam Government, Ha Noi, Vietnam; 3 Biomedical and Pharmaceutical Research Center, Vietnam Military Medical University, Ha Noi, Vietnam; 4 System Emotional Science, Graduate School of Medicine and Pharmaceutical Sciences, University of Toyama, 2630 Sugitani, Toyama, 930–0194, Japan; 5 Dong Nai General Hospital, Bien Hoa City, Dong Nai Province, Vietnam; 6 Department of Pediatric Neurology, Hyogo Children’s Sleep and Development Medical Research Center, Hyogo Rehabilitation Center Hospital, 1070 Akebono, Nishi-ku, Kobe, Hyogo, 651–2181, Japan; Institute for Health & the Environment, UNITED STATES

## Abstract

Dioxin concentrations remain elevated in the environment and in humans residing near former US Air Force bases in South Vietnam. Our previous epidemiological studies showed adverse effects of dioxin exposure on neurodevelopment for the first 3 years of life. Subsequently, we extended the follow-up period and investigated the influence of perinatal dioxin exposure on neurodevelopment, including motor coordination and higher cognitive ability, in preschool children. Presently, we investigated 176 children in a hot spot of dioxin contamination who were followed up from birth until 5 years old. Perinatal dioxin exposure levels were estimated by measuring dioxin levels in maternal breast milk. Dioxin toxicity was evaluated using two indices; toxic equivalent (TEQ)-polychlorinated dibenzo-p-dioxins/furans (PCDDs/Fs) and concentration of 2,3,7,8-tetrachlorodibenzo-p-dioxin (TCDD). Coordinated movements, including manual dexterity, aiming and catching, and balance, were assessed using the Movement Assessment Battery for Children, Second Edition (Movement ABC-2). Cognitive ability was assessed using the nonverbal index (NVI) of the Kaufman Assessment Battery for Children, Second Edition (KABC-II). In boys, total test and balance scores of Movement ABC-2 were significantly lower in the high TEQ- PCDDs/Fs group compared with the moderate and low exposure groups. NVI scores and the pattern reasoning subscale of the KABC-II indicating planning ability were also significantly lower in the high TCDD exposure group compared with the low exposure group of boys. However, in girls, no significant differences in Movement ABC-2 and KABC-II scores were found among the different TEQ-PCDDs/Fs and TCDD exposure groups. Furthermore, in high risk cases, five boys and one girl highly exposed to TEQ-PCDDs/Fs and TCDD had double the risk for difficulties in both neurodevelopmental skills. These results suggest differential impacts of TEQ-PCDDs/Fs and TCDD exposure on motor coordination and higher cognitive ability, respectively. Moreover, high TEQ-PCDDs/Fs exposure combined with high TCDD exposure may increase autistic traits combined with developmental coordination disorder.

## Introduction

During military operations by the US Armed Forces between 1961 and 1971, large quantities of herbicides were sprayed in Southern Vietnam. One of these herbicides, Agent Orange, contained 2,3,7,8-tetrachlorodibenzo-p-dioxin (TCDD), which is the most toxic dioxin congener of polychlorinated dibenzo-p-dioxins/furans (PCDDs/Fs). Several decades after spraying of herbicides, dioxin concentrations in the environment and in humans remain elevated in the sprayed areas of Vietnam. Particularly, former United States airbases located in Bien Hoa, Da Nang, and Phu Cat are hot spots of dioxin contamination owing to the huge amount of herbicide stored and spilled there during mixing and loading [1,2]. We recently reported that levels of TCDD and PCDDs/Fs in breast milk of mothers residing near hot spots were significantly higher compared with mothers living in unsprayed areas [3,4], suggesting perinatal dioxin exposure including high prenatal dioxin exposure by placental transfer from mothers and postnatal dioxin exposure through breastfeeding to their offspring. Since the brain is undergoing a variety of developmental processes during the perinatal period and is sensitive to neuro-toxins such as dioxins, prenatal exposure by maternal transfer may contribute to adverse neurodevelopmental effects in offspring which appear in later life [5].

Our previous epidemiological studies in a hot spot of dioxin contamination in Da Nang, Vietnam, showed that perinatal dioxin exposure indicated by dioxin levels in maternal breast milk which correlates with maternal blood levels [6,7], has considerable adverse effects on growth and neurodevelopment of children from birth to 3 years [8–11]. We found inverse associations between toxic equivalent (TEQ)-PCDDs/Fs and TCDD exposure and neurodevelopment of infants aged approximately 4 months [8], particularly in boys [9]. Inverse association between TEQ-PCDDs/Fs and TCDD exposure and social emotional scores were observed in toddlers at 1 year of age [10]. Moreover, we followed up these infants until 3 years old and reported that perinatal TCDD exposure increased autistic traits in boys and girls without effects on general neurodevelopment including cognitive, language, and motor function, while TEQ-PCDDs/Fs exposure only affected general neurodevelopment in boys [11].

These results suggested that perinatal dioxin exposure, particularly TCDD, might affect higher brain functions more than general neurodevelopment in children with neurodevelopmental disorders. Thus, to assess higher cognitive ability such as planning and face recognition, the Kaufman Assessment Battery for children, Second Edition (KABC-II) is a suitable candidate test for Vietnamese children as it has a nonverbal index validated for non-English-speaking children.

The Movement Assessment Battery for Children (Movement ABC) is a widely used test assessing motor skills in school-age children, and a high prevalence of coordination movement impairments has been reported in children with autism spectrum disorder (ASD). Green et al. (2008) assessed impairments in coordination movement skills, using the Movement ABC, and found definite movement impairments in 79% of children with ASD [12]. Ament et al. (2014) used the second edition of this battery (Movement ABC-2) to assess the specificity of coordination movement impairments in children with autism, reporting that catching and balance were significantly negatively correlated with developmental disability [13]. Therefore, Movement ABC-2 might be an appropriate assessment tool to examine coordination movement skills in children exposed to dioxins in Vietnam.

In the present study, we extended the follow-up period of preschool children in a hot spot of dioxin contamination in Da Nang, Vietnam, to 5 years after birth. In this cohort, we investigated the influence of perinatal dioxin exposure, indicated by breast milk concentration, on neurodevelopment, including motor coordination and higher cognitive ability, using the Movement ABC-2 and KABC-II tests.

## Materials and Methods

### Study Areas and Subjects

Da Nang city is located in the middle of Vietnam and Da Nang airbase is a known hot spot of dioxin contamination. The Thanh Khe and Son Tra districts in Da Nang City, located within 10 km of Da Nang airbase, were selected as the study locations. In collaboration with the Vietnamese government, Hatfield Consultants monitored environmental hazards, including heavy metals and organochlorines in the soil of the area surrounding Da Nang airbase, reporting that dioxins are prominent among the hazardous chemicals found there [2].

Enrolled subjects were children whose mothers were living in the Thanh Khe and Son Tra districts in Da Nang city. They were recruited at birth by obstetricians at district hospitals in Thanh Khe and Son Tra. The criteria for recruitment of mothers at the hospitals were as follows: i) mothers must have resided in one of the study districts for a period encompassing at least the duration of their pregnancy; ii) mothers must have given birth to full-term babies; and iii) there must have been no complications during childbirth. A total of 241 mother–infant pairs were enrolled at baseline, including 159 mothers that gave birth in the Thanh Khe district hospital in 2008 and 82 mothers that gave birth in the Son Tra district hospital in 2009. Subsequently, follow-up examinations were conducted at 4 months, 1 year, and 3 years after birth. Of 241 mother–infant pairs at baseline, 78.0% (188 pairs; 120 in Thanh Khe and 68 in Son Tra) participated in the 5-year follow-up assessment, conducted in Thanh Khe in December 2013 and in Son Tra in March 2015. Fifty-three pairs did not receive a follow-up assessment because they moved to other areas or were absent on test days. Of children who participated in the follow-up, mother’s information was missing for seven children. Thus, the final sample for the analysis included 181 children (115 and 66 children from Thanh Khe and Son Tra districts, respectively), which represented 75.1% of the original sample. No significant differences were found regarding characteristics or breast milk dioxin levels between participants and drop-outs, or included and excluded participants. Written informed consent to participate in the survey and to publish data was obtained from all mothers according to a process reviewed and approved by the Health Department of Da Nang City. The study design was approved by the Institutional Ethics Board for Epidemiological Studies at Kanazawa Medical University (No. 187).

### Interview and Growth Parameters

Information was collected from mothers (age, residential history, parity, smoking habits of the mother and other family members, alcohol consumption, education, and family income) and the children (age at the assessment, gestational weeks, and gender). Physical parameters of the children, including weight, height, and head and abdominal circumferences, were measured at birth and 5 years, and mean (SD) of measurement values are shown in Table 1.

**Table 1:** Characteristics of subjects in three groups with different exposure levels of TEQ-PCDDs/Fs in breast milk.

	TEQ-PCDDs/Fs groups	Low		Middle		High		
Gender		Mean	SD	Mean	SD	Mean	SD	P-value
Boys	Number of subjects	39		39		25		
	Maternal age	26.3	4.3	27.9	6.5	30.9	5.7	**
	Maternal education (years)	9.4	2.5	8.6	3.6	8.2	4.0	
	Family income (VNDsx10^3^/month)	3012	1620	3167	1462	2733	1420	
	Parity (primiparae rate (%))	15.4		34.2		29.2		
	Child age (months)	62.3	1.6	62.2	1.7	61.7	1.8	
	Gestational age at birth (weeks)	39.7	0.9	39.5	0.6	39.7	0.7	
	Birthweight (g)	3335	357	3220	384	3241	460	
	Weight (kg) at the survey	20.2	3.8	20.1	4.0	19.3	4.8	
	Height (cm) at the survey	109.1	3.5	109.4	4.4	103.7	19.6	
	Head circum (cm) at the survey	50.9	1.4	50.7	1.1	50.7	1.8	
	Abdominal circum (cm) at the survey	55.2	6.0	55.3	6.7	54.1	6.5	
	BMI at the survey	16.9	2.4	16.7	2.5	16.4	2.8	
	TCDD (pg/g lipid)	0.86	1.8	1.6	1.7	2.3	1.5	**
	TEQ-PCDDs/Fs (pg-TEQ/g lipid)	8.3	1.3	13.9	1.1	21.1	1.2	**
Girls	Number of subjects	26		26		26		
	Maternal age	27.0	5.2	27.9	6.4	31.0	6.6	
	Maternal education (years)	8.0	3.7	8.4	4.2	8.3	3.2	
	Family income (VNDsx10^3^/month)	2965	1745	2759	1294	2907	1822	
	Parity (primiparae rate (%))	23.1		34.8		22.2		
	Child age (months)	61.8	1.9	62.0	1.8	61.7	1.7	
	Gestational age at birth (weeks)	39.7	0.7	39.4	0.8	39.5	0.9	
	Birthweight (g)	3125	368	3096	350	3322	351	
	Weight (kg) at the survey	17.5	3.5	19.5	4.5	18.4	3.5	
	Height (cm) at the survey	107.7	4.6	109.6	4.4	106.8	3.9	
	Head circum (cm) at the survey	49.5	1.5	50.0	1.5	49.6	1.7	
	Abdominal circum (cm) at the survey	51.6	5.2	54.4	7.5	53.3	6.4	
	BMI at the survey	15.0	2.2	16.1	2.9	16.0	2.3	
	TCDD (pg/g lipid)	0.62	2.3	1.6	1.9	3.3	1.7	**
	TEQ-PCDDs/Fs (pg-TEQ/g lipid)	7.2	1.4	14.4	1.1	22.6	1.3	**

High TEQ-PCDDs/Fs group: TEQ-PCDDs/Fs ≥ 17.6pg-TEQ/g lipid, Moderate TEQ-PCDDs/Fs group: 11.5–17.6, Low TEQ-PCDDs/Fs group: <11.5

SD: standard deviation, VND: Vietnam Dong, Head circum: Head circumference, Abdominal circum: Abdominal circumference

Mean and SD of dioxin concentrations are geometric ones.

**: P<0.01: compared with low TEQ-PCDDs/Fs group

### Breast Milk Collection and Measurement of PCDDs/Fs in Breast Milk

A breast milk sample was collected from each nursing mother 1 month after birth by hand expression with the assistance of a midwife or medical worker. Samples were placed in clean polyethylene containers and temporarily stored at − 4°C in local health centers. Samples were frozen on dry ice for transport to Japan by airplane, and were analyzed at the Center for High Technology, Kanazawa Medical University (Uchinada, Japan). Approximately 10 mL of breast milk was used to quantify levels of 17 2,3,7,8-substituted PCDDs/Fs, including seven congeners of PCDDs and 10 congeners of PCDFs, using a gas chromatograph (HP-6980; Hewlett-Packard, Palo Alto, CA, USA) equipped with a high-resolution mass spectrometer (MStation-JMS700, JEOL, Tokyo, Japan). The methods and results of dioxin analysis have been described previously [3, 8]. Briefly, 17 congeners of PCDDs/Fs were quantified and concentrations in picograms per gram of lipid (pg/g lipid) were calculated. TEQ of PCDDs/Fs was calculated as the sum of all values obtained, multiplying each congener concentration by its TEQ factor from the WHO 2005-TEF [14]. Values of congeners with concentrations below detection limits were set to half the detection limit.

Subjects were divided into three groups (high, moderate, and low exposure groups) according to TCDD and TEQ-PCDDs/Fs levels in breast milk as follows: a) TCDD low <2.5, medium 2.5–3.5, and high ≥3.5 pg/g lipid; and b) TEQ-PCDDs/Fs low <11.5, medium 11.5–17.6, and high ≥17.6 pg-TEQ/g lipid. For analysis of KABC-II scores in children aged 60 months or older, the subjects were divided into two groups according to the TCDD in breast milk with a cutoff level of 2.5 pg/g lipid.

### Neurodevelopmental Assessments

In the present study, we used two neurodevelopment assessment batteries for children, Movement ABC-2 and KABC-II. Children were tested in a community health station by one examiner for each test in the presence of the child’s caregiver. The examiners were blinded to exposure levels and previous neurodevelopmental outcomes of each subject. During the Movement ABC-2 testing, 5 children refused to cooperate and the final sample for analysis of coordination movement included 176 children, although all children were participated KABC-II.

Movement ABC-2 (Pearson Education Inc., UK) is a measure of manual dexterity (MD), aiming and catching (A&C), and balance (Bal) of children over 3 years old. The MD scale assesses skills such as posting coins, threading beads, and drawing trails. The A&C scale assesses skills such as catching beanbags and throwing beanbags onto mats. The Bal scale assesses skills such as one-leg balance, walking with heels raised, and jumping on mats. For a total MD score, we added the three subscale scores, for a total A&C score, we added the two subscale scores. For the total Bal score, we added the three Bal subscale scores. Finally, we added each total score to obtain a total test score (TOTAL).

Cognitive abilities of the children were assessed using KABC-II (NCS Pearson Inc., USA). Cognitive functions used in the present study included conceptual thinking, face recognition, number recall, triangles (shape matching), word order, pattern reasoning, and hand movements. Nonverbal index (NVI), sequential/global scales of short-term memory (Seq/Gsm), and simultaneous/global scales of visual processing (Sim/Gv) were used as global scales. Scores for conceptual thinking, face recognition, triangles, pattern reasoning, and hand movements for 5-year-olds, and scores for four of these subscales excluding pattern reasoning for 4-year-olds were combined to provide a NVI score. Number recall and word order scores were summed to provide a score of Seq/Gsm. Finally, Sim/Gv score was the total score of conceptual thinking, triangles, and pattern reasoning tests for 5-year-olds, with the latter replaced by face recognition for 4-year-olds.

### Data Analysis

SPSS software (Version 21.0,; IBM Corp., Armonk, NY, USA) was used for statistical analyses. The concentrations of dioxins in breast milk were base-10 logarithmically transformed to improve normality. A general linear model was used to compare Movement ABC-2 and KABC-II scores of the three TEQ-PCDDs/Fs or TCDD exposure groups after adjusting for the following covariates: parity (primipara or multipara), maternal age, family members’ smoking habits, family income (Vietnamese Dong), maternal alcohol consumption during pregnancy (yes or no), maternal education (years), gestational age at birth, birth weight, age at present examination (months), and area of residence (Thanh Khe or Son Tra). Single variable tests were used to test significant difference among exposure groups. Results were considered significant if P <0.05.

## Results

### Comparisons of Subject Characteristics among TEQ-PCDDs/Fs Exposure Groups

Among the 176 mother-children pairs participated in neurodevelopmental assessments at 5 years old, we compared the mean and standard deviation (SD) of characteristics between the three TEQ-PCDDs/Fs exposure groups. The results split by gender are shown in Table 1. In both boys and girls, there was no significant difference in the characteristics of mothers or children among the three TEQ-PCDDs/Fs exposure groups, except for maternal age, which was significantly higher in the high TEQ-PCDDs/Fs group compared with the low exposure group. Levels of TCDD were significantly increased with increasing levels of TEQ-PCDDs/Fs in these three groups (Table 1).

### Comparisons of Movement ABC-2 Scores among TEQ-PCDDs/Fs and TCDD Exposure Groups

Table 2 shows the adjusted Movement ABC-2 scores in the three TEQ-PCDDs/Fs exposure groups for each gender; high (≥17.6 pg-TEQ/g lipid), moderate (11.5–17.5 pg-TEQ) and low (<11.5) exposure groups. In boys, the TOTAL score was significantly lower in the high TEQ-PCDDs/Fs exposure group than in the moderate and low exposure groups. For subscales, total BAL score was significantly lower in the high TEQ-PCDDs/Fs group compared with the moderate and low exposure groups in boys. Among the three BAL scores, scores for jumping on mats contributed to the decreased total BAL score for boys in the high TEQ-PCDDs/Fs exposure group. The score for walking with heels raised was also lower in the high TEQ-PCDDs/Fs exposure group, but this difference in scores was exhibited only between the moderate and high TEQ-PCDDs/Fs exposure groups in boys. In girls, no significant differences in Movement ABC-2 scores were found among the three TEQ-PCDDs/Fs exposure groups. Additionally, there was no significant difference in Movement ABC-2 scores among the three TCDD exposure groups in children of any gender (data not shown).

**Table 2:** Comparisons of adjusted Movement ABC-2 scores among the three TEQ-PCDDs/Fs exposure groups.

	TEQ-PCDDs/Fs groups	Low		Moderate		High		Group cpmparison		ANOVA	Effect size
Gender	Scales	Mean	SE	Mean	SE	Mean	SE	High-Low	High-Mod	P-val	
Boys	Number of subjects	39		38		24					
	Total test score (TOTAL)	9.7	0.4	9.8	0.4	7.7	0.5	**	##	0.018	0.107
	Total manual dexterity (MD)	10.1	0.5	10.8	0.5	9.5	0.6			0.262	0.033
	Posting coins (MD1)	8.8	0.4	8.8	0.4	7.7	0.5			0.198	0.040
	Threading beads (MD2)	9.8	0.5	11.7	0.5	10.0	0.6			0.031	0.074
	Drawing trail (MD3)	10.2	0.4	10.2	0.4	10.1	0.5			0.981	0.003
	Total aiming & catching (A&C)	9.9	0.4	9.4	0.4	9.7	0.5			0.712	0.012
	Catching beanbag (A&C1)	9.8	0.3	9.6	0.3	9.9	0.4			0.819	0.006
	Throwing beanbag (A&C2)	9.5	0.5	8.9	0.5	8.9	0.6			0.679	0.011
	Total balance (BAL)	9.3	0.4	9.2	0.4	7.3	0.5	**	##	0.013	0.094
	One-leg balance (BAL1)	9.5	0.4	9.0	0.4	8.5	0.5			0.419	0.041
	Walking heels raised (BAL2)	7.7	0.4	8.0	0.4	6.5	0.5		#	0.112	0.037
	Jumping on mats (BAL3)	11.0	0.4	11.6	0.4	9.5	0.5	*	##	0.010	0.085
Girls	Number of subjects	26		23		26					
	Total test score (TOTAL)	10.1	0.5	9.9	0.5	9.9	0.5			0.959	0.002
	Total manual dexterity (MD)	10.2	0.5	10.4	0.5	10.5	0.5			0.935	0.006
	Posting coins (MD1)	8.0	0.6	8.8	0.6	8.5	0.6			0.646	0.022
	Threading beads (MD2)	11.1	0.6	11.1	0.6	10.8	0.6			0.923	0.001
	Drawing trail (MD3)	10.7	0.4	9.7	0.4	10.9	0.4			0.140	0.058
	Total aiming & catching (A&C)	9.5	0.4	9.4	0.4	9.7	0.4			0.835	0.004
	Catching beanbag (A&C1)	10.1	0.4	9.4	0.4	9.4	0.4			0.324	0.042
	Throwing beanbag (A&C2)	8.2	0.6	8.8	0.6	9.5	0.6			0.330	0.025
	Total balance (BAL)	10.7	0.7	10.7	0.7	10.0	0.7			0.738	0.020
	One-leg balance (BAL1)	9.4	0.5	9.1	0.5	9.9	0.5			0.550	0.021
	Walking heels raised (BAL2)	9.2	0.7	8.6	0.7	9.2	0.7			0.775	0.010
	Jumping on mats (BAL3)	11.2	0.4	11.8	0.4	11.3	0.4			0.528	0.025

High TEQ-PCDDs/Fs group: TEQ-PCDDs/Fs ≥ 17.6pg-TEQ/g lipid, Moderate TEQ-PCDDs/Fs group: 11.5–17.6, Low TEQ-PCDDs/Fs group: <11.5

MD1-3: Subscales of MD, A&C1-2:Subscales of A&C, BAL1-3; Subscales of BAL

ANOVA P-value: compared by General Linear Model after adjustment for month-age, gestational age, birth weight of infant, family income, parents' education years.

*: P<0.05,

**: P<0.01 comparison between high and low TEQ-PCDDs/Fs groups,

^#^: P<0.05,

^##^: P<0.01 comparisons between high and moderate TEQ-PCDDs/Fs groups

### Comparisons of KABC-II Scores among TEQ-PCDDs/Fs and TCDD Exposure Groups

Table 3 shows comparisons of adjusted KABC-II scores among the high (≥ 3.5 pg/g lipid), moderate (2.5–3.5 pg/g lipid), and low (< 2.5 pg/g lipid) TCDD groups. In boys, NVI scores were significantly lower in the high TCDD exposure group compared with the low exposure group, although there was no significant difference in NVI scores in girls. There were no significant differences in Seq/Gsm and Sim/Gv scores among the three TCDD exposure groups in either gender. Subsequently, NVI scores were compared between the three TCDD exposure groups in children of 5 years or older, as NVI score is assessed differently in 5-year-olds, including the pattern reasoning assessment. In boys, NVI scores in 5-year-olds were significantly lower in the high TCDD exposure group compared with the low exposure group (Table 3). However, there were no significant differences between TCDD exposure groups in Seq/Gsm and Sim/Gv scores in boys or in any scores in girls. Additionally, no significant differences in cognitive global scores were found between the three TEQ-PCDDs/Fs exposure groups in all children or the subgroup of 5-year-olds of any gender (data not shown).

**Table 3:** Comparisons of adjusted KABC-II scores among the three TCDD exposure groups.

TCDD groups			Low		Moderate		High		Comparison	ANOVA	Effect size
Age	Gender	Scales	Mean	SE	Mean	SE	Mean	SE	High-Low	P-val	
All age	Boys	Number of subjects	85		11		7				
		NVI	91.8	1.6	83.8	4.5	77.0	6.2	*	0.034	0.076
		Seq/*Gsm*	102.7	1.8	109.0	5.1	103.1	7.0		0.515	0.014
		Sim/*Gv*	96.4	2.2	84.6	6.3	86.6	8.6		0.163	0.037
	Girls	Number of subjects	57		10		11				
		NVI	94.7	2.1	94.7	5.1	98.1	5.0		0.818	0.006
		Seq/*Gsm*	109.5	2.2	106.2	5.3	105.6	5.2		0.739	0.008
		Sim/*Gv*	94.0	2.4	92.6	5.7	99.2	5.6		0.647	0.008
5-year-olds	Boys	Number of subjects	74		10		6				
		NVI	89.7	1.6	81.4	4.4	74.0	6.4	*	0.024	0.093
		Seq/*Gsm*	102.5	1.9	107.6	5.2	101.5	7.3		0.634	0.010
		Sim/*Gv*	94.9	2.3	92.1	6.4	85.8	9.0		0.151	0.043
	Girls	Number of subjects	47		10		11				
		NVI	94.3	2.4	93.5	5.3	96.7	5.2		0.892	0.004
		Seq/*Gsm*	108.9	2.6	107.3	5.7	108.5	5.4		0.969	0.003
		Sim/*Gv*	92.1	2.7	93.3	6.0	101.0	5.7		0.392	0.018

High TCDD group: TCDD ≥ 3.5 pg/g lipid, Moderate TCDD group: 2.5–3.5, Low TCDD group: < 2.5,

SE: standard error, N: number of subjects, NVI: Non Verbal Index,

Seq/Gsm: Sequence/General ability of short term memory, Sim/Gv: Simultaneous/General ability of visual processing

ANOVA P-value: compared by General Linear Model after adjustment for month-age, gestational age, birth weight of infant, maternal age, family income, parents' education years.

*: P<0.05: compared with low TCDD group

To compare KABC-II subscale scores, 5-year-olds were divided into two groups: low (< 2.5 pg/g lipid) and high (≥ 2.5 pg/g lipid). The cutoff of 2.5 pg/g lipid was used, because the number of boys with TCDD ≥ 3.5 pg/g lipid was small. In boys, NVI scores were significantly lower in the high TCDD exposure group compared with the low exposure group (Table 4). Boys in the high TCDD group also had lower Sim/Gv scores, but this difference was not significant (P = 0.056). Among subscales of KABC-II scores, the pattern reasoning contributed to the decreased NVI score in the high TCDD exposure group (Table 4). In boys, face recognition scores were lower in the high TCDD exposure group, but this difference was not significant. These results suggest that decreased pattern reasoning scores were the biggest contributor to decreased NVI scores in boys.

**Table 4:** Comparisons of adjusted KABC-II scores between high and low TCDD exposure groups in 5-year-olds.

		Low		High			
(pg/g lipid)		TCDD < 2.5		TCDD ≥ 2.5			
Gender	Scales	Mean	SE	Mean	SE	P-value	Effect size
Boys	Number of subjects	74		16			
	NVI	90.1	1.6	78.9	3.8	0.010	0.083
	Seq/*Gsm*	102.5	1.9	105.8	4.3	0.508	0.006
	Sim/*Gv*	86.6	1.7	78.3	3.9	0.065	0.043
	Sub 1: Conceptual Thinking	6.8	0.32	5.8	0.74	0.244	0.017
	Sub 2: Face Recognition	8.8	0.27	7.6	0.63	0.099	0.035
	Sub 3: Triangles	10.0	0.35	9.3	0.81	0.434	0.008
	Sub 4: Pattern Reasoning	7.1	0.33	5.3	0.79	0.041	0.052
	Sub 5: Hand Movements	9.9	0.23	9.2	0.53	0.255	0.017
	Sub 6: Number Recall	11.4	0.37	11.8	0.85	0.678	0.002
	Sub 7: Word Order	9.3	0.35	10.0	0.81	0.476	0.007
Girls	Number of subjects	47		21			
	NVI	94.3	2.4	95.3	3.8	0.839	0.001
	Seq/*Gsm*	109.3	2.5	107.5	4.0	0.721	0.002
	Sim/*Gv*	88.6	2.5	92.6	3.9	0.420	0.012
	Sub 1: Conceptual Thinking	6.8	0.46	7.4	0.73	0.521	0.007
	Sub 2: Face Recognition	9.4	0.44	10.6	0.69	0.191	0.003
	Sub 3: Triangles	10.8	0.41	10.5	0.65	0.639	0.004
	Sub 4: Pattern Reasoning	7.1	0.53	8.7	0.85	0.132	0.040
	Sub 5: Hand Movements	10.4	0.36	9.2	0.58	0.117	0.043
	Sub 6: Number Recall	13.0	0.48	13.2	0.76	0.872	0.000
	Sub 7: Word Order	10.1	0.49	9.3	0.78	0.428	0.011

SE: standard error, Sub: subscale, NVI = Sub 1–5, Seq/Gsm = Sub 6+7, Sim/Gv = Sub 1+2+4

P-value: compared by General Linear Model after adjustment for month-age, gestational age, birth weight of infant, maternal age, family income, parents' education years

### Comparison of the Effects of TEQ-PCDDs/Fs and TCDD on Neurodevelopment

To investigate the relationship between dioxin exposure and neurodevelopmental impairments, children were divided into three risk groups for impairment according to the number of risks present for difficulties in motor coordination and/or cognitive function (no risk, single risk of either function, or double risk of both functions). We compared the rate of children with TEQ-PCDDs/Fs ≥17.6 pg-TEQ/g lipid and TCDD ≥2.5 pg/g lipid in the three risk groups for neurodevelopmental impairment (Table 5). We used the guidebook for each assessment to define cutoff values for being at risk of neurodevelopmental impairment. For the Movement ABC-2, this was the 15^th^ percentile of the general population (67 points) [15]. For the NVI scale, this was -2SD of the general population (70 points) [16]. Five boys (5%) and one girl had double risks for difficulties in both neurodevelopmental skills, while 25 boys (25%) and 12 girls (15.8%) had a single risk for impairment in either motor coordination or cognitive skill. In boys, children at double risk had 100% higher exposure rates for TEQ-PCDDs/Fs exposure and 80% for TCDD exposure. These rates were significantly higher compared with children at no risk for impairment (Table 5). In boys at single risk, only the rate of TEQ-PCDDs/Fs ≥17.6 pg-TEQ/g lipid was significantly higher compared with children at no risk (Table 5). Additionally, 23 boys (92%) at single risk had difficulties in motor coordination. However, none of these effects were found for girls.

**Table 5:** Children at risk for having difficulties in motor coordination and/or cognitive ability.

	Number of risk	0	1	2
Gender	Dioxin levels	N (%)	N(%)	N(%)
Boys	Number of subjects	71	25	5
	TEQ-PCDDs/Fs ≥ 17.6 (pg-TEQ/g lipid)	10 (14.1)	6 (24.0)	5 (100.0)
	TCDD ≥2.5 (pg/g lipid)	10 (14.1)	3 (12.0)	4 (80.0)
Girls	Number of subjects	63	12	1
	TEQ-PCDDs/Fs ≥ 17.6 (pg-TEQ/g lipid)	22 (34.9)	1 (8.3)	0 (0.0)
	TCDD ≥2.5 (pg/g lipid)	17 (27.0)	3 (25.0)	0 (0.0)

N: number of children

## Discussion

### Effects of Dioxin on Motor Coordination and Higher Cognitive Abilities

In the present study, scores for motor coordination, particularly balance, were significantly lower in preschool boys exposed to high levels of TEQ-PCDDs/Fs during the perinatal period, although movement impairment was not associated with TCDD exposure. In contrast, NVI scores (indicating cognitive ability) were lower in boys exposed to high levels of TCDD, but not TEQ-PCDDs/Fs. In the present subjects, the most contributed congeners to TEQ-PCDDs/Fs in breast milk were 1,2,3,7,8-PentaCDD (34%), 2,3,4,7,8-PentaCDF (18%), and 1,2,3,6,7,8-HexaCDF (14%), while the contribution rate of TCDD was 12% of TEQ-PCDDs/Fs. These congeners, particularly 2,3,4,7,8-PentaCDF, have different toxic effects compared with TCDD in human tissue or cells [17, 18]. Thus, the effects of TEQ-PCDDs/Fs on motor coordination might be caused by other PCDDs/Fs congeners than TCDD. However, boys at double risk for both movement and cognitive difficulties tended to be those with higher exposure to both TEQ-PCDDs/Fs and TCDD, suggesting that high levels of total dioxin exposure with increased TCDD may increase the risk of cognitive impairment combined with motor coordination problems similar to children with ASD [12,13].

### Impact of Dioxin Exposure on Motor Development in Previous Studies

In previous assessments of the present cohort at 3 years old, we reported that motor scores (assessed using the Bayley III instrument), cognitive measures, and language scores were all lower in boys exposed to high TEQ-PCDDs/Fs (≥17.6 pg-TEQ/g lipid) [11]. It is possible that boys in the high TEQ-PCDDs/Fs exposure group who showed poor general motor skills at 3 years old, also exhibited poor motor coordination skills at five years old, suggesting that TEQ-PCDDs/Fs exposure increases the risk for developmental coordination disorder (DCD). Indeed, children with DCD often show delays in achieving motor milestones when they are younger [19]. The Movement ABC is a widely used test of motor skills for school-age children, and is also used to detect motor outcomes of children exposed to environmental pollutants. Roze et al. (2009) administered the Movement ABC, version 1, to 62 children in the Netherlands to investigate the influence of prenatal exposure to organohalogen compounds, such as hydroxylated polychlorinated biphenyls (OH-PCBs), on motor development. They reported an inverse correlation between OH-PCBs and fine manipulation abilities (i.e. a low MD score) [20]. However, balance control, which was particularly affected by TEQ-PCDDs/Fs exposure in the present study, is a sensorimotor integration skill often impaired in children with ASD [21–24]. These motor effects may arise from a common mechanism in the brain, as high TCDD exposure has previously been shown to increase autistic traits at 3 years of age [11]. However, some boys at single risk for movement difficulties were exposed to high levels of TEQ-PCDDs/Fs but not high TCDD. High TEQ-PCDDs/Fs exposure without increased TCDD could occur in any areas exposed to dioxins from causes other than Agent Orange. These results suggest that such exposure might increase the risk of motor coordination difficulties specifically. This might be caused by alteration of reaction time of visual processing due to impairment of myelination in the central nervous system [25]. Our results provide a new perspective on adverse health effects of perinatal dioxin exposure in preschool children, as increased risk for DCD resulting from dioxin exposure has not previously been reported.

### Impact of Dioxin Exposure on Higher Cognitive Ability in Children: Present and Previous Studies

To examine cognitive abilities of children, we used the NVI of KABC-II to derive a total global scale score, because only this scale was validated for a non-English-speaking population. Among the five subscales of the NVI for 5-year-olds, pattern reasoning scores contributed most to decreased NVI scores in boys exposed to high TCDD (≥2.5 pg/g lipid). This suggests that poor planning ability is related to increased TCDD exposure. Scores for face recognition were also lower in boys in the high TCDD exposure group, although the difference in scores was not significant compared with low TCDD groups. These findings suggest that poor cognitive ability observed in the present subjects might be similar to that in children with ASD, who have difficulties in planning and face recognition [26]. This speculation is also supported by the result that TCDD exposure did not affect Seq/Gsm scores, including number recall and word order, both of which are poor in children with mental retardation. In our previous study of the present subjects at 3 years old, we reported increased autistic traits in the high TCDD exposure group (TCDD ≥ 3.5 pg/g lipid) in both genders [11]. However, in the present study conducted at 5 years of age, boys in the higher TCDD group (with a lower cutoff value of 2.5 pg/g lipid) showed increased risk for ASD-like cognitive deficits. Since testing for higher cognitive abilities is more appropriate at 5 than at 3 years old, we suggest that we have detected mild effects of dioxin exposure on cognitive ability in boys exposed to moderately increased TCDD. Future investigations are required to investigate whether the present cohort shows deficits similar to those in children with ASD in higher cognitive abilities, such as executive functions, including planning and theory of mind [27].

A Dutch cohort study of school-age children reported an inverse relationship between dioxin exposure and both visual motion processing and cognition [28], but no relationship between dioxin exposure and neurodevelopmental test results, including IQ [25]. This suggests that effects of dioxin exposure on cognitive functions in children might only be evident in specific higher brain functions. Consequently, they can only be detected by specific psychological tests and neurophysiological examinations, but not by general developmental tests. Therefore, we are planning to follow-up these children and assess alterations in specific higher cognitive functions, using neurophysiological tests such as EEG responses to visual and auditory stimuli.

### Effects of Dioxins on Higher Brain Function and Mechanisms in Animal Studies

In animals, we reported that prenatal TCDD exposure induces socio-emotional deficits particularly in male offspring owing to alterations in Ca^2+^/calmodulin-dependent protein kinase IIα (CaMKIIα) activity and decreased number of parvalbumine-positive neurons (putative inhibitory interneurons) in the limbic system of rat brains [29,30]. These results suggested that perinatal TCDD exposure influences social brain function, a higher brain function, that is also observed in children with ASD. Moreover, Endo et al. (2012) reported behavioral inflexibility, compulsive repetitive behavior, and lower competitive dominance in mice exposed to TCDD during the perinatal period using IntelliCage apparatus and suggested that perinatal TCDD exposure may lead to develop executive function deficits and social behavioral abnormality [31]. In rat offspring exposed to TCDD during the perinatal period, Kakeyama et al. (2014) also demonstrated poor paired-associate learning behavior, suggesting that perinatal TCDD exposure disrupted higher brain function [32]. These higher brain function deficits were indicated to be accompanied with alteration of neuronal activity in the medial prefrontal cortex and amygdala of the limbic system of mice brains [31]. These reports are consistent with our finding of lower cognitive scores in 5-year-old children with high TCDD exposure in the present study. Further studies are required to determine the exact neuronal alterations in the brain, and effects on neuronal networks, particularly limbic system of the brain.

### Gender Difference

Consistent with our previous studies of the birth cohort in Da Nang at 4 months [9] and 3 years of age [11], gender differences in the effects of dioxin on neurodevelopment were evident in the present study. Our results suggest that boys are more susceptible to dioxin toxicity than girls. Higher prevalence of ASD and DCD in boys is also well established [20, 33]. The interaction between genes and the environment leading to early exposure to androgenic hormones is a potential etiological mechanism affecting sex-specific susceptibility to ASD [34]. An inverse association between testosterone levels in cord blood and maternal di (2-ethylhexyl) phthalate exposure, a persistent organic pollutant, was reported in a Japanese birth cohort [35]. These results suggest that maternal dioxin exposure might influence testosterone levels of the fetus which play an important role in autistic traits in childhood.

A study in the Netherlands reported endocrine disrupting effects of perinatal PCDDs/Fs and PCBs exposure on play behaviors exhibited by children, with more feminized play observed in exposed boys at 8 years of age [36]. Recently, Winnike et al. (2014) also investigated sex-typical play behaviors of German children aged 6–8 years and reported that perinatal exposure to PCDDs/Fs and PCBs might modify behavioral sexual dimorphism in children [37]. They suggested that dioxins interact with the hypothalamic-pituitary-gonadal axis in the brain to induce endocrine-disrupter effects. In the same German cohort, it was also reported that prenatal and perinatal exposure to PCDDs/Fs and PCBs influenced attention-deficit hyperactivity disorder—related behavior [38]. These results suggest that male susceptibility to the effects of dioxin on neurodevelopment might be mediated through an interaction of dioxins with the hypothalamic-pituitary-gonadal axis, consequently affecting androgenic hormone levels in the fetal brain. Our future studies following up these children will investigate associations between dioxin exposure and sexual dimorphism, in addition to cognitive abilities.

### Limitations

The validity of the results and conclusions of the present study must be considered within the context of the study’s strengths and limitations. Although the Movement ABC-2 and KABC-II are useful tests for making independent quantitative judgments regarding motor coordination and cognitive ability, they have some limitations with respect to the present study. The Movement ABC-2 was developed and standardized for infants in the United Kingdom, not for Vietnamese children. Therefore, we could not judge the developmental levels of individual children based on the scores obtained with this test. However, we used their cutoff value to assess risk for movement difficulties, resulting in possible overestimation of children with possible DCD. Regarding KABC-II, it is possible that we overestimated the number of children at risk for cognitive impairment, albeit we used the NVI, which is validated for non-English-speaking populations. However, each test was performed by a single examiner who was well trained and followed specific instructions as described in the manual. Therefore, the comparison of test scores among the different exposure groups within the same population, to clarify associations between the levels of dioxin exposure and test scores of the test should be reliable.

## Conclusions

These results demonstrate the considerable impact of perinatal dioxin exposure on neurodevelopment in boys at 5 years of age. However, TEQ-PCDDs/Fs and TCDD exposure differently affected neurodevelopment. TEQ-PCDDs/Fs increased the risk of disability in motor coordination, while TCDD increased the risk of higher cognitive deficits. Furthermore, high TEQ-PCDDs/Fs exposure combined with high TCDD exposure may increase autistic traits combined with DCD, particularly in boys. A longer follow-up study of the present cohort in Vietnam is required to clarify whether the effects of dioxin on neurodevelopment will be evident in school-age children.

## Supporting Information

S1 Dataset Minimum data of the study. (XLSX)

S1 Text Abbreviations. (DOCX)
