# Supplementary material for: Impacts of Perinatal Dioxin Exposure on Motor Coordination and Higher Cognitive Development in Vietnamese Preschool Children: A Five-Year Follow-Up
Source: PLoS One. 2016 Jan 29;11(1):e0147655. doi: 10.1371/journal.pone.0147655 (PMC4732982; doi:10.1371/journal.pone.0147655)
Supplement: S1 Text — (DOCX) [file pone.0147655.s002.docx]

**Abbreviations**

TCDD: 2,3,7,8-tetrachlorodibenzo-p-dioxin

1,2,3,7,8-PentaCDD: 1,2,3,7,8-pentachlorodibenzo-p-dioxin

2,3,4,7,8-PentaCDF: 2,3,4,7,8-pentachlorodibenzofuran

1,2,3,6,7,8-HexaCDF: 1,2,3,6,7,8-hexachlorodibenzofuran

PCDDs: polychlorinated dibenzo-p-dioxins

PCDFs: polychlorinated dibenzofurans

PCDDs/Fs: polychlorinated dibenzo-p-dioxins/furans

TEQ: toxic equivalent

TEF: toxic equivalent factors

Movement ABC-2: Movement Assessment Battery for Children, Second Edition

KABC-II : Kaufman Assessment Battery for Children, Second Edition

ASD: autism spectrum disorder

DCD: developmental coordination disorder
